# PowerScope 2 functional appliance: A 3D finite element simulation of its action on the mandible

**DOI:** 10.1016/j.jobcr.2023.02.014

**Published:** 2023-03-01

**Authors:** Afrah Khazal Al Hamdany, Lamiaa A. Hasan, Mohammed Najeeb Abdullah Alrawi, Emad Hazim Kasim Alhajar

**Affiliations:** a Department of Pedodontics, Orthodontics, and Prevention /College of Dentistry/Mosul University, Iraq; b Department of Mechanical Engineering, College of Engineering, University of Mosul, Mosul, Iraq

**Keywords:** Finite element, Functional appliance, Orthodontics, PowerScope 2

## Abstract

PowerScope 2 is a fixed functional appliance for patients with Class II malocclusion and a retrognathic mandible, that has recently received attention due to its pronounced advantages, for both orthodontists and patients.

**Objective:**

of study: This study evaluated the action of the PowerScope 2 appliance for correcting Class II malocclusion and the stresses and displacement of the mandible during loading using three-dimensional finite element analysis (FEA). The sites of the mandibular skeletal and/or dental corrections were also distinguished.

**Materials and methods:**

Using the AutoCAD (2010) Program, a 3D model of the human mandible with teeth was created based on a CT image of a 20-yr-old patient**.** Orthodontic stainless-steel brackets with Standard Edgewise (0.022 in) slots bonded to five mandibular teeth and inserted into a bounded tube on the first molar were simulated. A rectangular archwire (0.019 × 0.025 in) ligated the brackets. The created models were uploaded to the Autodesk Inventor Professional Computer Program (FE) version (2020).

**Results:**

The FEA presented the three-dimensional results qualitatively and quantitatively as von Mises stress and displacement. The colour ruler on the upper left side demonstrates the stress and displacement distribution pattern of the mandible, with the minimum value in blue and the maximum value in red. Mandibular movement was achieved three-dimensionally. There was obvious sagittal forward mandibular movement, and high stress was observed at the chin prominence (the pogonion). In the transverse plane, the mandible was highly bent buccally, especially at the gonial angle and antegonial notch. In the vertical plane, the highest ranges of mandibular movements were seen in the chin, the anterior part of the mandibular body, and the associated dentoalveolar region.

**Conclusions:**

The results of this FEA, PowerScope 2 functional appliance proved to be effective as a Class II malocclusion corrector. Its mode of action on the mandible was achieved in three planes of space, and its orthodontic effects were gained dentally and skeletally. An bbvious sagittal forward mandibular movement was observed, particularly at the chin prominence. Apparent buccal bending, especially at the gonial angle and antegonial notch, was observed. Vertically, the chin and anterior part of the mandible, with the associated dentoalveolar structures, were clearly stressed under the action of this appliance.

## 1 Introduction

In Germany (1909), Emil Herbst designed an appliance for the early correction of Class II malocclusion. The appliance was a removable splint with an inclined plane for forward mandibular movement.^1^ The correct use of such removable appliances by patients was often questionable, irrespective of patient compliance. Thus, Herbst designed a fixed appliance to move the mandible forward. Subsequently, many different fixed functional appliances to correct Class II malocclusion were developed.^2^

Based on 20 years of clinical experience in the use of fixed functional appliances for Cl II malocclusion in Brazil, Moro et al. described the development of their functional appliances and highlighted their properties.^3^ There is a widespread scope for fixed functional appliances. Therefore, is not easy to select an optimal option for each patient. Based on the forces used to move the mandible forward, Portuguese orthodontists, Ritto and Ferreira,^2^ classified different functional appliances into flexible, rigid, and hybrid types. Flexible fixed functional appliances consist of an intermaxillary coil spring or fixed spring.^2^ These appliances, such as the Jasper Jumper, and Jasper Vector, are elastic and flexible, which allows for an appropriate extent of free mandibular movements and easy lateral guidance.^4^ Rigid fixed functional appliances, such as the Herbst appliance,^5^ mandibular protractor appliance (MPA), and mandibular anterior repositioning appliance (MARA), differ from the flexible types in that they resist fracture. However, rigid fixed functional appliances are not flexible or elastic. Once fitted and activated, they hinder the patient's habitual maximal intercuspation.^2^ However, they produce greater skeletal effects than flexible types.^6^ The hybrid fixed functional appliances, such as the Forsus, Twin Force, and PowerScope, have features of both flexible and rigid appliances. The hybrid type is characterised by a spring system^2^ for moving teeth using continuous elastic force 24 h daily. The spring system replaces Class II elastics. Open springs are typically used to produce a range of forces (150 g–260 g) in this type of appliance. Notably, the chief indication for such appliances is not to produce a forward mandibular position. In a systematic review and meta-analysis by Ishaq et al.^7^ and Zymperdikas et al.,^8^ greater tooth movement than mandibular movement was observed during treatment with flexible or hybrid appliances. This may be attributed to the lack of movement of the condyle at the glenoid fossa,.^2^ In recent years, hybrid functional appliances have been used commonly. Typical characteristics of these newer functional appliances are a spring implanted within the telescopic system and the small size, thus improving patient comfort (e.g., protection against cheek pain and prevention of food accumulation) and adaptation.^9^

PowerScope^10^, ^11^, ^12^ is a relatively new hybrid fixed function appliance. The PowerScope is the latest innovative Class II functional appliance introduced by American Orthodontics, in association with Dr. Andy Hayes.^10^ According to the manufacturer, this functional appliance offers less chairside time, a simple manufacturing process and attachment, and minimal adjustments, which are advantageous for both patients and orthodontists ^(^10^)^. Since the PowerScope was first introduced in 2014, it has undergone three modifications (e.g. magnet key, stop reinforcement, and activation indicator piece). Currently, the most updated appliance is the PowerScope 2.

**Fig. 1:**
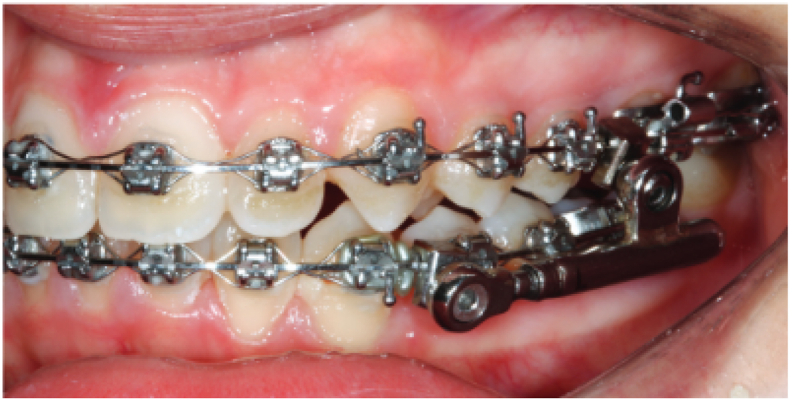
PowerScope 2 functional appliance (Moro et al., 2018).

The PowerScope appliance comprises a telescopic system with three durable fitting parts. It is a one-size-fits-all appliance that aids in controlling and saving space. A nickel-titanium spring generates a force of 260 g and represents its internal mechanism). Furthermore, wire-to-wire connections are possible, thus, permitting rapid and simple installation.^3^ Tubes that are bonded or welded to the molar bands are also present. Attachment nuts and hex screws are available at the ends, with the former being accountable for maintaining the telescopic system of the fixed appliance on the arch. The screw is secured using an Allen Hex key wrench. The attachment nut has a slot locked with a screw thread. Thus, the screw forms an inferior fourth surface to capture the wire when tightening. The formed slot is 0.020 × 0.026 in, in size. Notably, maxillary molar distalisation is easier with the PowerScope 2 because the system slides freely through the maxilla. In the mandible, this system slides similarly, but because of the curvature of the arch, it does not end at the canine bracket. Therefore, bracket detachment is decreased. Stainless steel archwires are used in both the maxilla and mandible. For 0.022 in slot brackets, a wire 0.019 × 0.025 in is used, while for the 0.018 in slots, a 0.017 × 0.025 in wire is used. To activate the appliance, spacers may be needed initially, depending on the severity of the Class II malocclusion and teeth sizes (Fig. 1). The spring of the PowerScope 2 is completely activated, irrespective of the amount of movement (3 mm or 6 mm). The spring is reactivated every month until 1 mm or 2 mm of overcorrection of the buccal segment is achieved.^3^

## 2 Objective of the study

The exact mode of action of the PowerScope 2 appliance, especially on the mandible, has not been previously investigated. Thus, this study aimed to evaluate the action of the PowerScope 2 functional appliance for correcting Class II malocclusion. In particular, the stress and displacement of the mandible during loading were assessed via 3D FEA to distinguish the sites of mandibular skeletal and/or dental corrections.

## 3 Materials and methods

**Fig. 2:**
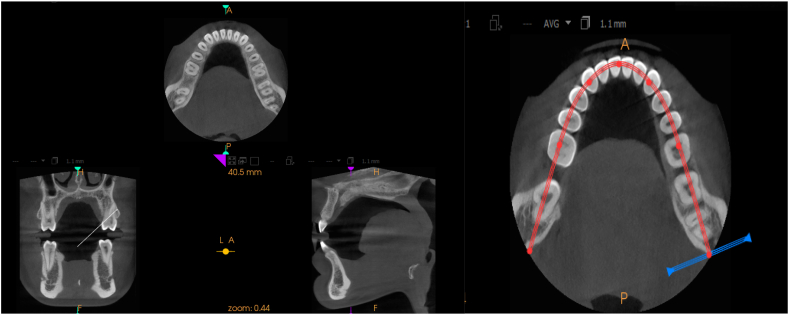
CT image for 3D model creation.

**Fig. 3:**
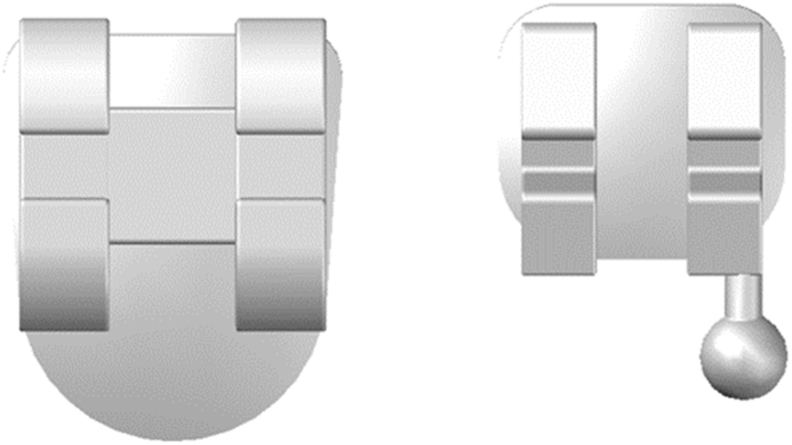
Bracket drawing within Auto CAD (2010).

**Fig. 4:**
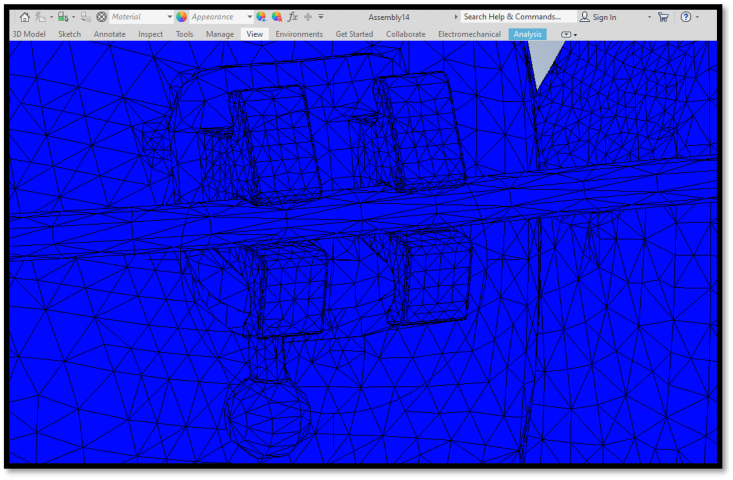
Bracket bonded on tooth surface.

Using the AutoCAD (2010) program,^13^ a 3D solid model of the mandible with teeth was created, based on a CT image of a 20-yr-old patient^14^ (Fig. 2). Scan data were obtained using multi-slice CT (Brilliance 64 slice, Philips Company, Amsterdam, Holland). The CT machine was set to 120 kV, 80 mA, exposure time 30 s, and 0.5-mm nominal slice thickness. Due to modelling complications and the difficulty of mesh generation and mathematical simulation, the periodontal ligaments were omitted. A network representing the cancellous bone was also ignored. Instead, the cancellous bone was represented as a solid unit within a shell of cortical bone.^15^,^16^ Standard orthodontic Edgewise stainless-steel brackets with 0.022 in slots on the five mandibular teeth and bounded tube on the first molar were simulated according to the Dentarum Orthodontics Catalogue Edition 22, (Fig. 3, Fig. 4). A rectangular archwire (0.019 × 0.025 in) ligated the brackets.^3^ The models were uploaded to the FE program, Autodesk Inventor Professional (2020). As suggested by many studies, the mechanical characteristics of the materials are isotropic, homogenous, and linear elastic.^15^^,^^16^ For isotropic materials, Young's modulus and Poisson's ratio were used as elementary inputs. Young's modulus and Poisson's ratio for the cortical bone, cancellous bone, teeth, stainless steel brackets, and wires, were adopted, as shown in Table 1.^17^

**Table 1:** Mechanical properties of materials.

Material	Young's modulus (MPa^a^)	Poissons Ratio
Cortical bone	13,000	0.3
Cancellous bone	1000	0.3
Teeth	20,000	0.3
Bracket	193,000	0.31
Wire	193,000	0.31

a MPa = Mega Pascal.

In this study model for simulation, the boundary condition was the outer surface of the mandibular condyle. The mandible was reinforced by a simulated glenoid fossa, which impeded the total movement at the top of the mandibular condyle.^16^^,^^17^ The auto-mesh order was used for mesh creation, and the accuracy of the result was affected by the number of elements elaborated in the model. The final mesh of this study model comprised 1 230 103 nodes and 803 419 elements. To simulate the PowerScope 2 appliance, a force between 150 g and 260 g were loaded mesially and vertically in relation to the distal surface of the mandibular first premolar.^3^

## 4 Results

Finite element analysis (FEA) qualitatively and quantitatively demonstrated the three-dimensional results, which were presented as von Mises stress and displacement. A colour ruler on the upper left side demonstrated the stress and displacement distribution pattern of the mandible; the minimum value appeared in blue. The maximum value is shown in red. Table 2 revealed the maximum von Mises stress and displacement values for both models.

**Table 2:** Values of maximum Von mises stress and displacement.

Variables	150 g	260 g
Von mises (Mpa^a^)	42.62	73.87
X-Displacement (mm^b^)	0.02601	0.04509
Y-Displacement (mm)	0.005092	0.008827
Z-Displacement (mm)	0.04999	0.08666

a (MPa) = Mega Pascal.

b (mm) = millimeter.

### 4.1 von mises stress

**Fig. 5:**
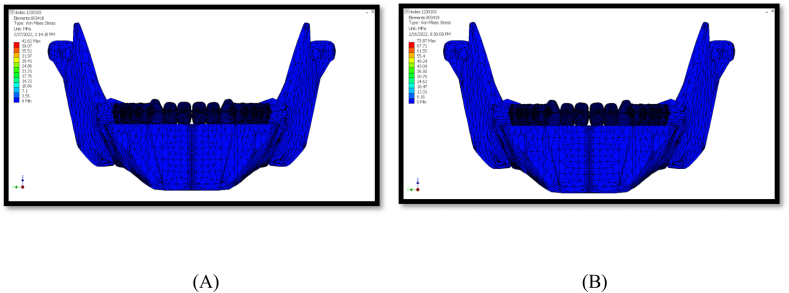
Von mises stress (A) for 150 g, (B) for 260 g.

For 150 g force, the maximum stress value was 42.62 MPa. For 260 g force, the maximum value was 73.87 MPa. Both conditions had the same pattern of stress distribution. The stress was concentrated close to the fixation areas and force application sites (Fig. 5).

### 4.2 Displacement

**Fig. 6:**
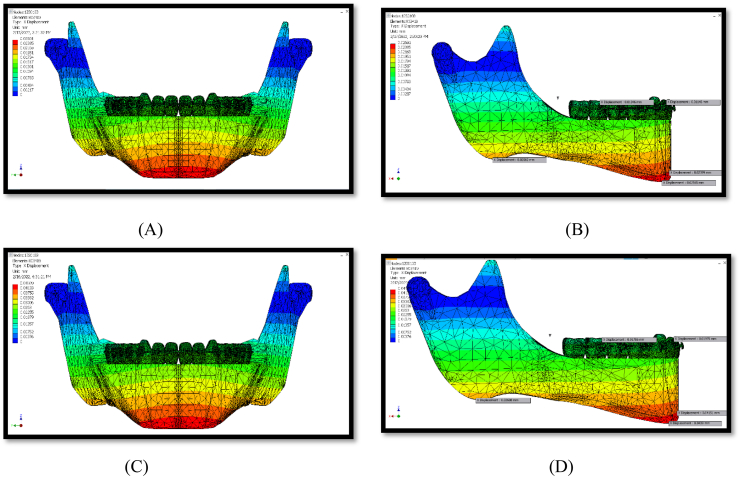
Displacement at x-axis (A, B) for 150 g, (C, D) for 260 g.

**Fig. 7:**
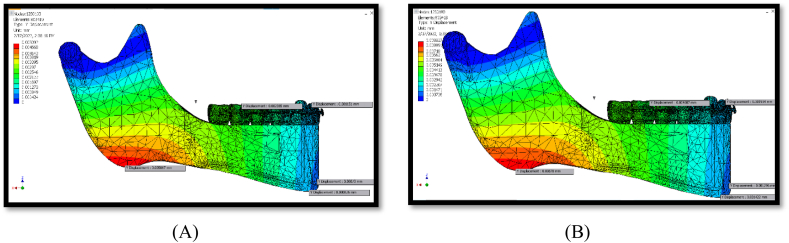
Y-displacement (A) for 150 g, (B) 260 g.

**Fig. 8:**
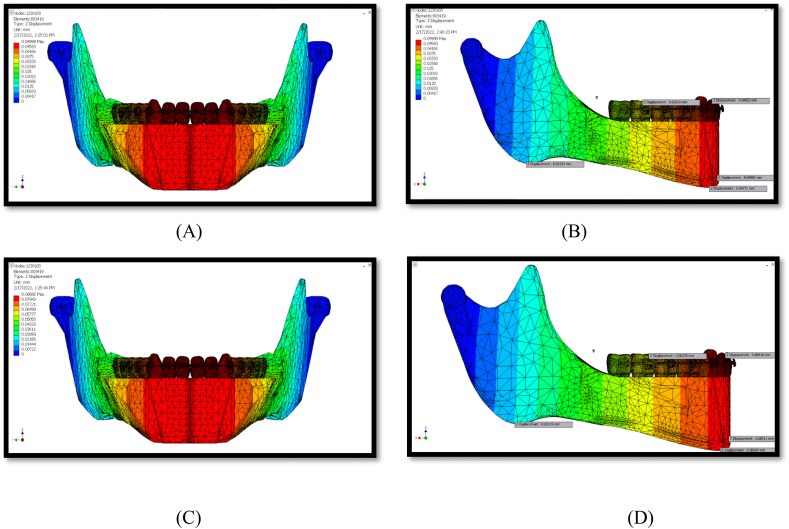
Z- Displacement (A, B) for 150 g, (C, D) for 260 g.

The maximum displacement was observed in the Z-direction (vertical movements). The lowest displacement value was observed in the Y direction (transverse) (Table 2). Both conditions exhibited the same displacement pattern, as shown in Fig. 6, Fig. 7, Fig. 8.

Using the “prob” command in the FEA (Autodesk Inventor Professional computer program), displacement was studied in selected anatomical landmarks to understand the pattern of displacement of the mandible under forces exerted by the PowerScope 2 appliance (Table 3).

**Table 3:** Displacement values for anatomical land marks.

Land marks	X-displacement (mm)		Y-displacement (mm)		Z-displacement (mm)	
	150 g	260 g	150 g	260 g	150 g	260 g
Central incisor edge	0.01146	0.01975	0.001151	0.001994	0.04852	0.08418
Pogonion (Pog)	0.02399	0.04151	0.00073	0.001296	0.04905	0.08511
Menton (Me)	0.02565	0.0439	0.000826	0.001422	0.04771	0.08284
Mesiobuccal cusp tip of first molar	0.01046	0.01786	0.0002385	0.0004007	0.03619	0.06378
Gonion (Go)	0.02082	0.03608	0.005067	0.00878	0.01224	0.02115

### 4.3 X-displacement

X-displacement illustrates the anteroposterior mandibular movements. Forces from the PowerScope 2 appliance caused the mandible to move forward. In particular, the most significant forward mandibular movement occurred at the chin prominence (the pogonion). The middle range of anterior movements was noticed within the area extending sagittally from the gonion point posteriorly to the anterior mandibular region above the chin area, and the least displacement was observed within the central region and the ascending ramus, as shown in Fig. 6.

### 4.4 Y-displacement

Y-displacement identifies the transverse movements. Forces exerted on the mandible by the Powerscope 2 appliance resulted in significant buccal tilting, especially at the gonial angle and antegonial notch of the mandible. The middle range of transverse movements was recognised within the middle and distal areas of the mandibular body and in the middle part of the ascending ramus region above the gonial angle. The least transverse displacement was seen within the chin and the associated dentoalveolar region, the uppermost part of the ascending ramus, and the coronoid processes (Fig. 7).

### 4.5 Z-displacement

Z-displacement identifies the vertical movements. Forces from the PowerScope 2 appliance led to the most significant vertical movements in the chin, the anterior part of the mandibular body, and the associated dentoalveolar region. The middle range of vertical movement was detected within the premolar and molar areas. The least vertical movement was observed within the gonial angle and the ascending ramus areas (Fig. 8).

## 5 Discussion

The PowerScope 2 is a new functional appliance that is gaining acceptance for the treatment of patients with a Class II malocclusion and a retrognathic mandible.^18^ Its advantages include a design that is well accepted by patients, no laboratory work, and less chairside time. It is prefabricated and ready to use chairside. The PowerScope 2 has a simple attachment system and a durable telescopic mechanism, an internal spring system of NiTi that diminishes the treatment time, together with a ball and socket joint system that exploits lateral mandibular movements for patient comfort.^10^

Previous reports on the PowerScope 2 functional appliance in the mandible are limited. Only a few studies have investigated the effectiveness of the PowerScope appliance in the treatment of patients of Class II division 1.

FEA is a useful tool for the prediction and calculation of the effects of stress applied in certain directions and at certain areas, and the stress distribution in the surrounding bone (cortical or cancellous) during different interventions.^19^

The findings of this study were consistent with the results of previous studies that investigated the effectiveness of the PowerScope 2 appliance for the treatment of Class II malocclusion. In particular, using three-dimensional FEA, the stresses and displacement of the mandible during loading were found to be more or less similar to previous studies.^21^, ^22^, ^23^ Mandibular movements were achieved in three planes of space. There were obvious sagittal forward mandibular movements, with high stress seen within the chin prominence area (the pogonion). In the transverse plane, the mandible was highly tilted buccally, especially at the gonial angle and antegonial notch. In the vertical plane, the most significant movement was seen in the chin, the anterior part of the mandibular body, and the associated dentoalveolar region.

This study differed from previous studies in design. For example, previous studies have included adolescents with different Class II malocclusions, diagnostic records, malocclusion severity, treatment duration, and/or skeletal and dentoalveolar outcomes.

The results from a case report by Paulose et al.^20^ concurred with the findings of this study. Paulose et al. illustrated the efficiency of the PowerScope in a 13-yr-old patient for the correction of skeletal Class II with mandibular deficiency during active growth, using a non-extraction approach rather than contemplating extractions. This case was initially treated with fixed appliance, followed by the PowerScope. A comparison of pre-, mid-, and post-treatment cephalograms showed stable changes and successful results, with substantial improvement in the skeletal jaw relationship (e.g. significant forward displacement of the mandible) and an enhanced facial profile.

In 2018, Arora et al.^21^ compared the outcomes of the PowerScope and Forsus in the treatment of Class II Div 1 adolescents with late skeletal maturity. They found that the PowerScope group achieved greater forward mandibular molar and incisor movements and more marked dentoalveolar changes than the Forsus group. In another study, Antony et al.^20^ evaluated the amount, time, and rate of molar correction and efficacy of the PowerScope in patients, aged 15–19 years, with Class II malocclusion, and found that the rate of molar correction was greater than that reported in previous studies. Statistically significant changes were observed in the dentoalveolar parameters (e.g. mandibular molar advancement, mandibular incisor proclination, and reduction in overbite and overjet). Skeletally, owing to the anterior mandibular position, there were also statistically significant changes. Moreover, owing to the forward movement of the soft tissue pogonion, a significant improvement in the facial profile was observed. Another study by Malhotra et al.^24^ assessed the effects of the PowerScope in adolescent patients and found that there was a significant increase in mandibular length, and that the changes were the results of dentoalveolar and skeletal alternations. In particular, Malhotra et al. demonstrated increased effective mandibular lengths, achieved total molar correction, and reduced the overjet.

Singaraju et al.^18^ in their cephalometric study showed that the PowerScope can be used as a propeller for sagittal mandibular correction in patient with skeletal Class II Div 2, as the PowerScope was effective in providing skeletal and dental changes in both jaws, with the amount of skeletal alteration being 1.5 times higher than that found in other functional appliance studies. There was also a significant mesial shift in the mandible. Effective dentoalveolar changes, with significant proclination of the mandibular central incisors and mesial movement of the mandibular molar, were noted. The authors attributed the effects of functional appliances to muscular forces produced by distension of the musculature that holds the mandible forward.^25^ The dentoalveolar effects on the mandibular arch were due to less mesial movement of the lower molars associated with a greater proclination of the incisors, as reported in previous fixed functional appliance studies.^5^^,^^26^ Similarly, Kalra et al.^23^ studied and evaluated the dental, skeletal, and soft tissue changes associated with the 5-month use of the PowerScope appliance for growing patients with Class II malocclusion on a digital lateral cephalogram. Statistically significant skeletal changes including mandibular forward positioning, increased SNB angle with N perpendicular-Pogonion distance, improved Class II jaw-base relationship, reduction in Wit's appraisal, and ANB angle, were noted. Significant changes were also observed in the forward mandibular incisor positioning, distalisation, and intrusion of the maxillary molar, and reduction in overjet and overbite). Due to an increased labiomental angle, considerable improvement in the soft tissue facial profile was also observed.

### 5.1 The general mode of action and growth changes obtained by the PowerScope 2

In the current study, the analysis of the general growth changes associated with the PowerScope 2 showed that effective general growth could be increased and the chin position and anterior part of the mandibular body, as well as the associated dentoalveolar region, could be changed. These changes are most likely the result of condylar growth stimulation^27^^,^^28^ due to muscle stretching under the influence of the PowerScope 2. Additionally, the associated remodelling of the glenoid fossa and/or positional changes of the condyle within the glenoid fossa^27^^,^^29^ may also influence the amount and direction of effective condylar growth.

The significant vertical displacement of the chin and anterior part of the mandibular body, with the associated dentoalveolar region, noted with PowerScope 2 treatment was probably triggered by the treatment approach itself, as the PowerScope 2 displaced the mandible vertically along the incisal guidance path. Furthermore, the eruption of molars and premolars may also contribute to the increased vertical jaw growth.

### 5.2 Limitations

1. As with all FEA, false results in the adjacent areas due to the constraints of rigid fixation implied in the FE programs should be considered. In reality, these muscles permit extension to some degree. This represents the chief obstacle in modelling the natural boundary conditions in cFE studies.
2. Facial soft tissues, including the masticatory muscles, could not be simulated.

### 5.3 Conclusions

In this FE study, the PowerScope 2 functional appliance was effective for the treatment of patients with Class II malocclusion. The mode of action on the mandible was achieved three-dimensionally and the effects were gained from dental and skeletal alterations. Obvious sagittal forward mandibular movements were also observed, particularly at the chin prominence. Moreover, buccal tilting at the gonial angle and antegonial notch, was observed. Vertically, the chin, the anterior part of the mandible, and the associated dentoalveolar structures were clearly stressed under the actions of the PowerScope 2.
